# Regulatory T lymphocyte infiltration in metastatic breast cancer—an independent prognostic factor that changes with tumor progression

**DOI:** 10.1186/s13058-021-01403-0

**Published:** 2021-02-18

**Authors:** Jenny Stenström, Ingrid Hedenfalk, Catharina Hagerling

**Affiliations:** 1 Division of Clinical Genetics, Department of Laboratory Medicine, Lund University, SE-221 85 Lund, Sweden; 2 Division of Oncology, Department of Clinical Sciences Lund, Lund University, SE-221 85 Lund, Sweden

**Keywords:** Breast cancer, Metastasis, Immune cells, Regulatory T lymphocytes, FOXP3

## Abstract

**Background:**

Patients diagnosed with metastatic breast cancer have poor outcome with a median survival of approximately 2 years. While novel therapeutic options are urgently needed, the great majority of breast cancer research has focused on the primary tumor and less is known about metastatic breast cancer and the prognostic impact of the metastatic tumor microenvironment. Here we investigate the immune landscape in unique clinical material. We explore how the immune landscape changes with metastatic progression and elucidate the prognostic role of immune cells infiltrating primary tumors and corresponding lymph node and more importantly distant metastases.

**Methods:**

Immunohistochemical staining was performed on human breast cancer tissue microarrays from primary tumors (*n* = 231), lymph node metastases (*n* = 129), and distant metastases (*n* = 43). Infiltration levels of T lymphocytes (CD3^+^), regulatory T lymphocytes (Tregs, FOXP3^+^), macrophages (CD68^+^), and neutrophils (NE^+^) were assessed in primary tumors. T lymphocytes and Tregs were further investigated in lymph node and distant metastases.

**Results:**

T lymphocyte and Treg infiltration were the most clinically important immune cell populations in primary tumors. Infiltration of T lymphocytes and Tregs in primary tumors correlated with proliferation (*P* = 0.007, *P* = 0.000) and estrogen receptor negativity (*P* = 0.046, *P* = 0.026). While both T lymphocyte and Treg infiltration had a negative correlation to luminal A subtype (*P* = 0.031, *P* = 0.000), only Treg infiltration correlated to luminal B (*P* = 0.034) and triple-negative subtype (*P* = 0.019). In primary tumors, infiltration of T lymphocytes was an independent prognostic factor for recurrence-free survival (HR = 1.77, CI = 1.01–3.13, *P* = 0.048), while Treg infiltration was an independent prognostic factor for breast cancer-specific survival (HR = 1.72, CI = 1.14–2.59, *P* = 0.01). Moreover, breast cancer patients with Treg infiltration in their distant metastases had poor post-recurrence survival (*P* = 0.039). Treg infiltration levels changed with metastatic tumor progression in 50% of the patients, but there was no significant trend toward neither lower nor higher infiltration.

**Conclusion:**

Treg infiltration could have clinical applicability as a prognostic biomarker, deciphering metastatic breast cancer patients with worse prognosis, and accordingly, could be a suitable immunotherapeutic target for patients with metastatic breast cancer. Importantly, half of the patients had changes in Treg infiltration during the course of metastatic progression emphasizing the need to characterize the metastatic immune landscape.

**Supplementary Information:**

The online version contains supplementary material available at 10.1186/s13058-021-01403-0.

## Background

Advancements in diagnostic tools and new therapeutic options have resulted in earlier diagnosis and improved survival for patients with early breast cancer [1, 2]. However, patients developing metastatic breast cancer are still confronted with poor outcome [3, 4]. The current number of deaths from breast cancer is estimated to be over 600,000 per year worldwide, with metastatic disease accounting for the majority of deaths [5, 6]. The most common metastatic sites for breast cancer include metastasis to the liver, lung, and bone with a median survival of approximately 2 years [7, 8]. Importantly, the metastatic patterns differ between the various molecular breast cancer subtypes. The triple-negative (TN) subtype is associated with lower rates of metastases to the bone and a higher rate of metastases to the brain and lung as compared to luminal breast cancer [8, 9]. Different metastatic locations are also associated to different survival durations after established metastatic disease, with bone and loco-regional metastases having the most favorable prognosis [7, 10]. The underlying mechanism explaining differences in metastatic behavior and how the tumor microenvironment, including immune cells, affect the fate of metastatic tumors and the clinical outcome of metastatic breast cancer patients are largely lacking. The great majority of breast cancer research has focused on the primary tumor, while less is known about the metastatic tumor microenvironment, including the immune landscape, and its prognostic impact. Here we elucidate the prognostic role of T lymphocytes, macrophages, and neutrophils in primary tumors of patients with metastatic breast cancer. We further investigate T lymphocyte and regulatory T lymphocyte (Treg) infiltration in lymph node and distant metastases and examine how the immune landscape changes with metastatic progression.

T lymphocytes are part of the adaptive immune system. Previous studies have shown that a high number of total T lymphocytes, as well as high gene expression of the pan-T-lymphocyte marker CD3, in primary tumors are associated with better overall survival in breast cancer patients [11–14]. Infiltration of cytotoxic T lymphocytes has also been linked to increased survival in breast cancer [14–16]. Cytotoxic T lymphocytes also seem to play a role in metastatic breast cancer. Blake-Mortimer et al. showed that high levels of cytotoxic T lymphocytes in peripheral blood is linked to prolonged survival in patients with metastatic breast cancer [17].

Another important T lymphocyte subtype is Tregs. Tregs play a key role in regulating the immune response and possess the ability to suppress inflammatory responses [18]. In breast cancer, reports have shown an association between high Treg infiltration in primary tumors and shorter survival, which has also been concluded in meta-analyses [11, 19–21]. However, results seem to differ depending on the breast cancer subtype. For example, associations between Treg infiltration in primary tumors and better outcome have been demonstrated in estrogen receptor (ER)-negative breast cancer [22, 23]. Moreover, Tregs have been shown to play a role in the development of metastatic breast cancer. Olkhanud et al. were able to show that Tregs are important for the development of lung metastases in breast cancer mouse models [24]. Furthermore, Treg infiltration in primary tumors has been associated to the presence of circulating tumor cells in patients with breast cancer, suggesting a role in the dissemination of tumor cells [25].

Besides immune cells of the adaptive immune system, myeloid cells, such as macrophages and neutrophils and part of the innate immune response, have also been shown to have an important role in breast cancer development and prognosis. Several studies have found an association between high macrophage infiltration in primary breast cancer and poor prognosis [26–28]. Less is known about the role of neutrophils in breast cancer. However, Wculek et al. could in breast cancer mouse models demonstrate that neutrophils are important in promoting metastases to lungs [29].

Our study reveals the clinical importance of T lymphocytes, in particular Tregs, and their prognostic role in metastatic breast cancer and that their infiltration changes with metastatic progression.

## Material and methods

### Patient data and biological material

The breast cancer cohort analyzed initially included 304 women with metastatic breast cancer or locally advanced breast cancer taking part in a previous phase III clinical trial investigating different combinations of chemotherapy in metastatic breast cancer. The women were diagnosed with metastatic breast cancer in several different clinics in Sweden between 2002 and 2007. In brief, exclusion criteria were metastases to the brain, indication for HER2 therapy, or other diagnosed malignancy within 5 years from metastatic breast cancer diagnosis. The study cohort, construction of the tissue microarrays (TMAs), and evaluation of histopathological characteristics have previously been described in detail [30, 31]. In brief, primary tumor material was available from 231 patients. Corresponding lymph node metastases and distant metastases were assessed for 129 and 43 patients, respectively*.* Due to missing clinical information, pathological information, or low quality of TMA samples, fewer patients were included in the final analysis. The median age at diagnosis in the entire cohort was 50 years, ranging from 27 to 71 years of age. Median follow-up for patients alive at last follow-up was 8.2 years from the initial diagnosis.

### Immunohistochemistry

Immunohistochemistry (IHC) was performed on formalin-fixed paraffin-embedded tissue microarray (TMA) sections (4 μm). Slides were dried for 30 min in 60 **°**C and then deparaffinized in xylene before being rehydrated. The slides were blocked for endogenous peroxidase and then washed in PBS. Heat-induced epitope retrieval was then performed using Target Retrieval Solution (DAKO) in 95 °C in a pressure cooker heater for 20 min. Slides were then cooled down in RT for 20 min. The slides were washed in PBST, followed by removal of excess solution. For CD68, slides were protein blocked in Protein Block Serum-Free Solution (X0909, DAKO). Primary antibodies diluted in PBS containing 5% goat serum were applied. After 60 min in room temperature, slides were washed in PBST before the secondary antibodies were applied. Slides were incubated for 30 min in room temperature and then washed in PBS. Labeled substrate-chromogen solution was applied. One drop of Liquid DAB Chromogen (DAKO) was added for each 1 mL applied. Slides were then washed and counterstained in hematoxylin. Slides were rinsed in water followed by distilled water. To mount the slides, DAKO Paramount Aqueous Mounting Medium was used.

The following antibodies were used: rabbit monoclonal anti-CD3 (ab16669, Abcam (1:300)), mouse monoclonal anti-Foxp3 (ab20034, clone 236A/E7, Abcam, Cambridge, GB (1:400)), mouse monoclonal anti-CD68 (M0814, clone KP1, DAKO, Glostrup, Denmark (1:1400)), and rabbit anti-neutrophil elastase (NE) (ab68672, Abcam, Cambridge, GB (1:3000)). Secondary antibodies used included BrightVidion anti-mouse-HRP (DPVM55HRP, AH diagnostics, Tilst, Denmark), BrightVision anti-rabbit-HRP (DPVR55HRP, AH diagnostics), and Dako EnVision Dual Link System-HRP (K4063, Dako).

T lymphocytes (CD3+), Tregs (FOXP3+), macrophages (CD68+), and neutrophils (NE+) were assessed in primary tumor material. In lymph node and distant metastases, only T lymphocytes and Tregs were assessed. Each sample was scored from 0 to 3 depending on immune cell density for each immunohistochemical staining, 0 meaning no immune cells and 3 meaning a high infiltration density. From each patient, one to three tissue cores were available for evaluation in the TMA. The staining intensity was evaluated twice by J. S and C. H, blinded to all clinical information, first separately then combined interpretation.

### Statistical analysis

IBM SPSS Statistics version 26 was used for statistical analyses. Spearman’s rank correlation was used to explore the correlation between immune cell infiltration densities and clinicopathologic features. Clinicopathologic features which were included for primary tumors were age at diagnosis, T stage, nodal status, Ki67, histological grade, ER status, progesterone receptor (PR) status, and molecular subtypes. In analyses for lymph node metastases and distant metastases, age at diagnosis, Ki67, ER, PR, and molecular subtypes of lymph node and distant metastases were included.

Kaplan-Meier curves were constructed to compare survival in patients with different infiltration scores. Survival measurements included recurrence-free survival (RFS), breast cancer-specific survival (BCSS), and post-recurrence survival (PRS). Patients with advanced disease at initial diagnosis were excluded from RFS-analyses. Log-rank test was used to test significance. Analyses were performed with immune cell infiltration scores from 0 to 3, then dichotomized into low and high infiltration. T lymphocyte and macrophages were assigned low when scored 0, 1, or 2 and high when scored 3. Tregs and neutrophils were assigned low if scoring was 0 and high if scoring was 1, 2, or 3.

Cox regression proportional hazards models were used for estimation of hazard ratios (HRs) for recurrence and death from breast cancer according to T lymphocyte and Treg infiltration in both uni- and multivariable analysis. Covariates with a *P* value ≤ .05 in the univariable analysis were included in the multivariable analysis. All statistical tests were two sided and *P* ≤ .05 was considered significant.

McNemar’s test was used to investigate changes in T lymphocyte and Treg infiltration over metastatic progression. Dichotomized values for infiltration levels were used for this analysis.

## Results

### Immune cell infiltration in primary tumors

**Fig. 1:**
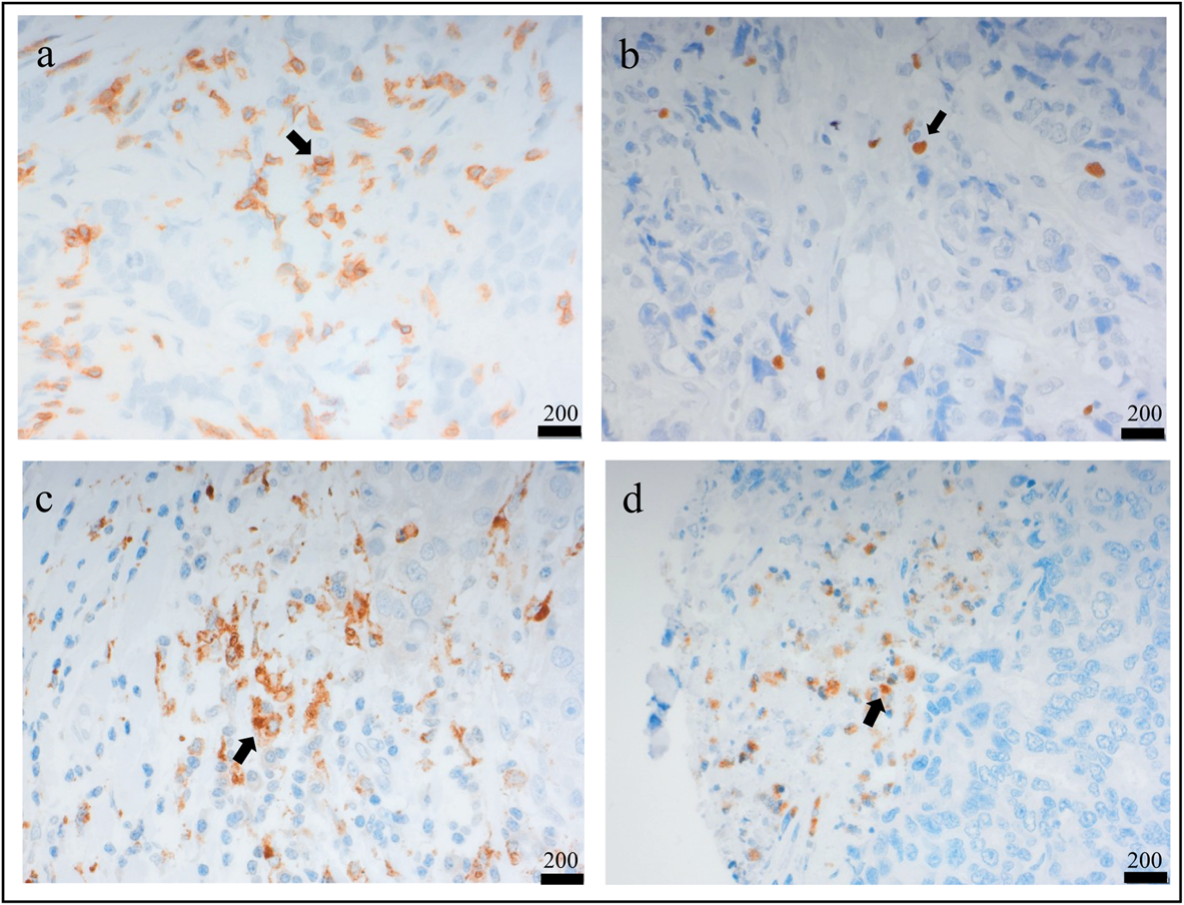
Immunohistochemical stainings of primary tumors. **a** CD3, a marker for T lymphocytes. **b** FOXP3, a marker for regulatory T lymphocytes. **c** CD68, a marker for macrophages. **d** NE, a marker for neutrophils. Scale bar = 200 μm. Black arrows indicate immune cells

**Fig. 2:**
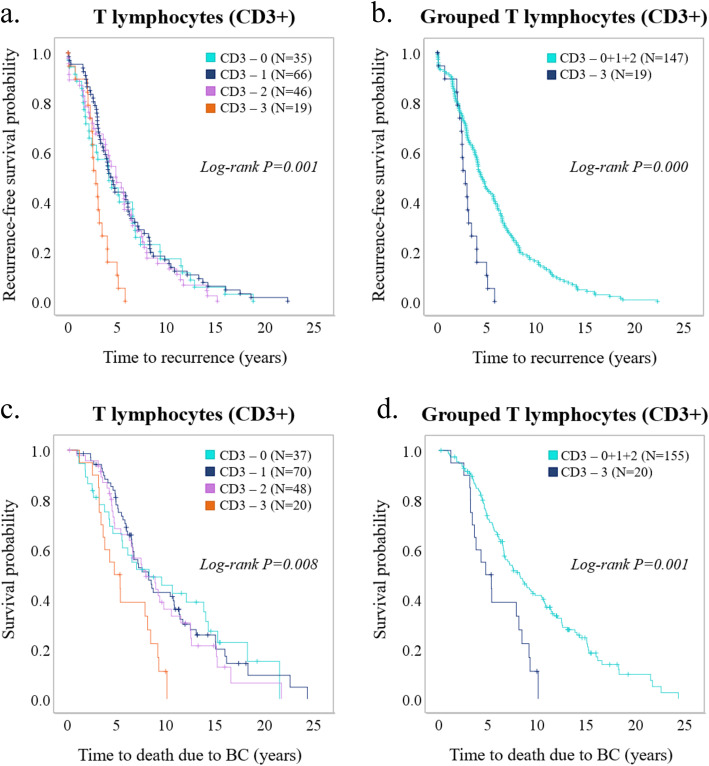
Prognostic role of T lymphocytes in primary tumors. Kaplan-Meier plots (log-rank test) for **a**, **b** recurrence-free survival and **c**, **d** breast cancer-specific survival among patients with different infiltration levels of T lymphocytes (CD3^+^) in primary tumors

We performed IHC on a TMA including 231 primary tumors from patients with metastatic breast cancer, to identify T lymphocytes (CD3^+^ cells), macrophages (CD68^+^ cells), and neutrophils (NE^+^ cells) (Fig. 1). T lymphocytes were shown to be the most clinically important immune cell population as compared to both macrophages and neutrophils (Fig. 2, Table 1, Additional files 1, 2, and 3). High T lymphocyte infiltration in primary tumors significantly correlated to several factors associated with poor prognosis, including high proliferation (Ki67 ≥ 20%) (*P* = 0.007) and negative ER status (*P* = 0.041). T lymphocyte infiltration also correlated inversely to luminal A subtype (*P* = 0.031) (Table 1). High T lymphocyte infiltration was associated to shorter recurrence-free survival (RFS) (*P* = 0.000) and breast cancer-specific survival (BCSS) (*P* = 0.001) (Fig. 2). T lymphocyte infiltration was a significant prognostic factor in univariable analysis for RFS (HR = 2.61, CI = 1.57–4.32, *P* = 0.000) and was an independent prognostic factor as shown by multivariable analysis (HR = 1.77, CI = 1.01–3.13, *P* = 0.048) (Table 2). For BCSS, T lymphocyte infiltration was significant only in univariable analysis (HR = 2.34, CI = 1.40–3.91, *P* = 0.001) (Table 3).

**Table 1:** Correlation between T lymphocyte (CD3+) and regulatory T cell (FOXP3+) infiltration and clinicopathological features in primary breast cancer

Variable	N (%)	CD3 infiltration				R	***P***	N (%)	FOXP3 infiltration				R	***P***
		**0**	**1**	**2**	**3**				**0**	**1**	**2**	**3**		
All	175 (100)	37	70	48	20			176 (100)	67	66	39	4		
Age														
< 50	79 (45.1)	15	36	22	6	0,04	0,60	81 (46.0)	30	29	19	3	-0,05	0,54
≥ 50	96 (54.9)	22	34	26	14			95 (54.0)	37	37	20	1		
Tumor size														
≤ 20 mm	73 (41.7)	17	28	20	8	0,03	0,71	73 (41.5)	30	27	15	1	0,06	0,42
> 20 mm	102 (58.2)	20	42	28	12			102 (58.0)	37	38	24	3		
Nodal metastasis														
-	57 (32.6)	13	22	17	5	0,03	0,74	57 (32.4)	16	26	13	2	-0,10	0,18
+	114 (65.1)	24	45	31	14			116 (65.9)	48	40	26	2		
Ki67														
-	108 (61.7)	28	47	23	10	0,21	**0,01**	109 (61.9)	52	38	18	1	0,33	**0,000**
+	59 (33.7)	9	19	21	10			58 (33.0)	10	25	20	3		
NHG														
Grade 1/2	62 (35.4)	12	31	16	3	0,06	0,43	64 (36.4)	27	24	13	0	0,06	0,48
Grade 3	91 (52.0)	22	32	24	13			91 (51.7)	34	36	17	4		
ER														
-	33 (18.9)	6	9	10	8	-0,16	**0,04**	32 (18.2)	8	11	12	1	-0,17	**0,03**
+	138 (78.9)	31	59	36	12			139 (79.0)	56	54	26	3		
PR														
-	74 (42.3)	18	23	20	13	-0,08	0,29	72 (40.9)	25	27	19	1	-0,07	0,39
+	96 (54.9)	19	45	26	6			98 (55.7)	39	38	18	3		
Luminal A	59 (33.7)	14	29	12	4	-0,17	**0,03**	60 (34.1)	32	20	7	1	-0,30	**0,000**
Luminal B	72 (41.1)	15	26	23	8	0,04	0,65	72 (40.9)	18	33	19	2	0,17	**0,03**
HER2	9 (5.1)	2	2	1	4	0,10	0,20	8 (4.5)	2	5	1	0	0,00	0,96
TN	22 (12.6)	3	7	9	3	0,11	0,16	22 (12.5)	5	6	10	1	0,18	**0,02**

*Abbreviations*: *N* number of patients included in analysis, *R* correlation coefficient, *Ki67* proliferation marker, *NHG* Nottingham histologic grade, *ER* estrogen receptor, *PR* progesterone receptor, *TN* triple negative. Spearman correlation, two-tailed *P* value. Bold indicates *P* value < 0.05

**Table 2:** Cox regression analyses for recurrence-free survival. Primary tumor

Variable	HR	(95% CI)	***P*** value	HR	(95% CI)	***P*** value
	Univariable			Multivariable		
Tumor size						
≤20 vs >20	1,52	(1.19 - 1.93)	**0,001**	1,39	(0.98 - 1.98)	0,068
NHG						
1, 2 vs 3	1,62	(1.19 - 2.19)	**0,002**	1,55	(1.07 - 2.25)	**0,021**
Nodal metastasis						
- vs +	1,31	(1.02 - 1.69)	**0,035**	1,71	(1.18 - 2.47)	**0,005**
ER						
- vs +	0,50	(0.33 - 0.74)	**0,000**	0,60	(0.37 - 0.97)	**0,037**
CD3						
low vs high	2,61	(1.57 - 4.32)	**0,000**	1,77	(1.01 - 3.13)	**0,048**
FOXP3						
low vs high	1,26	(0.91 - 1.74)	0,16			

*Abbreviations*: *HR* hazard ratio, *CI* confidential interval, *NHG* Nottingham histologic grade, *ER* estrogen receptor, *PR* progesterone receptor. CD3 indicates infiltration level of T lymphocytes (low 0, 1, 2 vs high 3) in primary tumor. FOXP3 indicates infiltration level of regulatory T lymphocytes (low 0 vs high 1, 2, 3). Spearman correlation, two-tailed *P* value. Bold indicates *P* value < 0.05

**Table 3:** Cox regression analyses for breast cancer-specific survival. Primary tumor

Variable	HR	(95% CI)	***P*** value	HR	(95% CI)	***P*** value
	Univariable			Multivariable		
Tumor size						
≤20 vs >20	1,49	(1.14 - 1.96)	**0,004**	1,41	(0.94 - 2.12)	0,094
NHG						
1, 2 vs 3	1,61	(1.14 - 2.28)	**0,007**	1,65	(1.07 - 2.53)	**0,023**
Nodal metastasis						
- vs +	1,51	(1.13 - 2.02)	**0,006**	2,01	(1.30 - 3.10)	**0,002**
ER						
- vs +	0,42	(0.27 - 0.63)	**0,000**	0,57	(0.34 - 0.97)	**0,038**
CD3						
low vs high	2,34	(1.40 - 3.91)	**0,001**	1,39	(0.77 - 2.50)	0,28
FOXP3						
low vs high	1,64	(1.39 - 2.37)	**0,008**	1,72	(1.14 - 2.59)	**0,010**

*Abbreviations*: *HR* hazard ratio, *CI* confidential interval, *NHG* Nottingham histologic grade, *ER* estrogen receptor, *PR* progesterone receptor. CD3 indicates infiltration level of T lymphocytes (low 0, 1, 2 vs high 3) in primary tumor. FOXP3 indicates infiltration level of regulatory T lymphocytes (low 0 vs high 1, 2, 3). Spearman correlation, two-tailed *P* value. Bold indicates *P* value < 0.05

Similar correlations to clinicopathological features were seen for macrophages and neutrophils (see Additional file 3). However, only macrophage infiltration had a prognostic impact, with high infiltration being associated with shorter BCSS (*P* = 0.037) consistent with prior research (see Additional files 1 and 2, [26–28]).

To better understand the immune landscape in breast cancer, we further investigated correlations between immune cell populations in primary tumors. We found significant correlations between T lymphocytes and Tregs (*P* = 0.000), T lymphocytes and macrophages (*P* = 0.000), T lymphocytes and neutrophils (*P* = 0.007), Tregs and macrophages (*P* = 0.000), and macrophages and neutrophils (*P* = 0.003) (see Additional file 4).

### Tregs in primary tumors—an independent prognostic factor

To further characterize which T lymphocyte subpopulation could potentially be of clinical importance, we decided to evaluate Tregs. Tregs in primary breast cancer have previously been shown to be associated with poor prognosis [11, 19, 20].

**Fig. 3:**
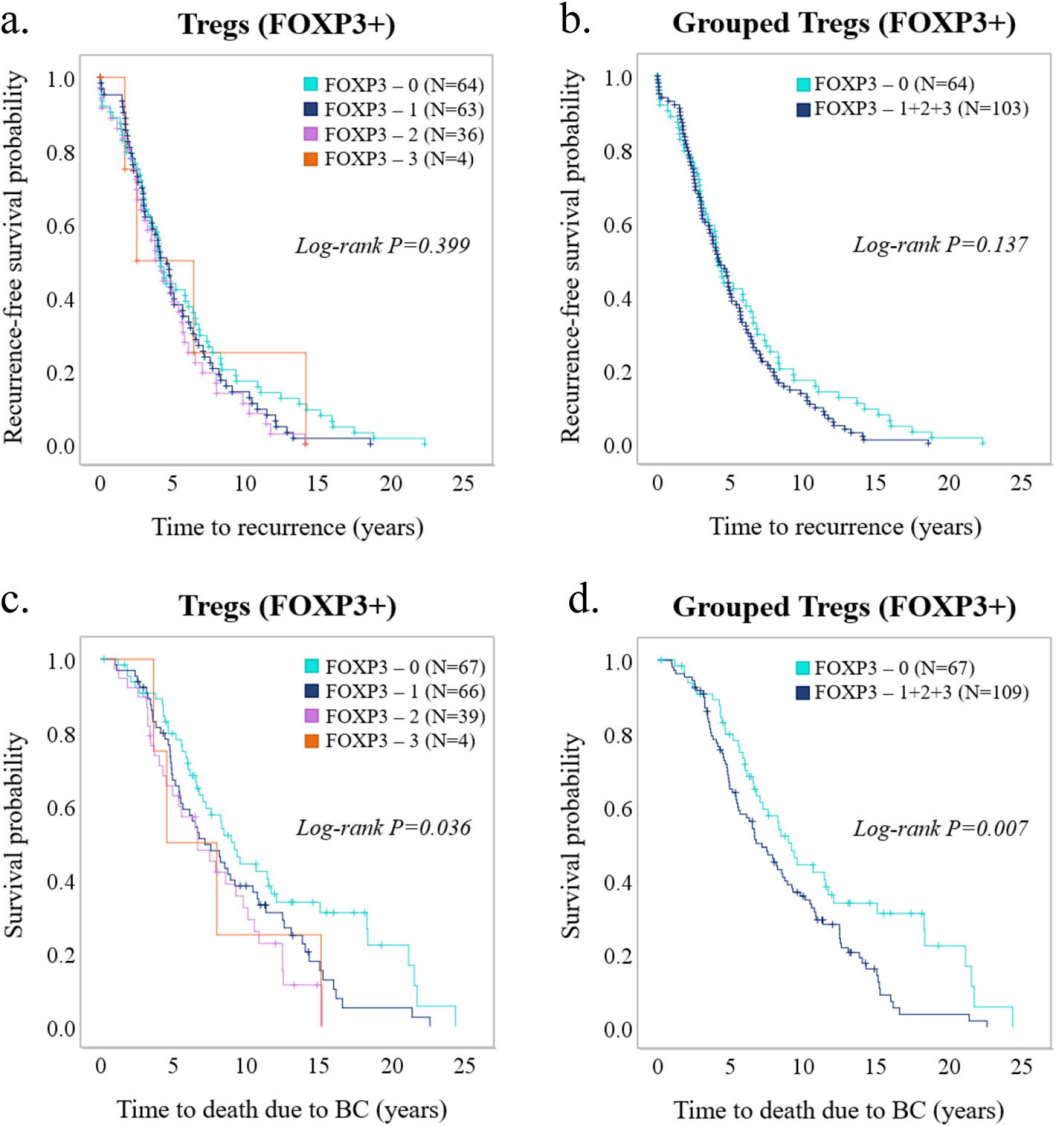
Prognostic role of Tregs in primary tumors. Kaplan-Meier plots (log-rank test) for **a**, **b** recurrence-free survival and **c**, **d** breast cancer-specific survival among patients with different infiltration levels of regulatory T lymphocytes (Tregs, FOXP3^+^) in primary tumor

Treg infiltration correlated to several prognostic factors, including high proliferation (*P* = 0.000) and negative ER status (*P* = 0.026). Moreover, high Treg infiltration correlated with luminal B (*P* = 0.034) and TN subtype (*P* = 0.019) and inversely to luminal A (*P* = 0.000) (Table 1). While Treg infiltration did not have any impact on recurrence, patients with high Treg infiltration showed worse BCSS (*P* = 0.007) (Fig. 3). Univariable analysis showed that patients with high Treg infiltration had a statistically significant increase in breast cancer-specific death rate (HR = 1.64, CI = 1.39–2.37, *P* = 0.008). Importantly, Treg infiltration was shown to be an independent prognostic factor for BCSS as determined with multivariable Cox regression analysis (HR = 1.72, CI = 1.14–2.59, *P* = 0.01) (Table 3).

### T lymphocyte and Treg infiltration changing with metastatic progression

In order to determine whether T lymphocyte and Treg infiltration would change or be unchanged over tumor progression, we evaluated their infiltration in all stages of tumor progression: primary tumors, lymph node, and distant metastases. While there were significant correlations between T lymphocyte and Treg infiltration within all stages of tumor progression, in primary tumor (*P* = 0.000), lymph node metastasis (*P* = 0.000), and distant metastasis (*P* = 0.000), there was no correlation between infiltration levels of T lymphocytes in primary tumors and distant metastases (Table 4). Discordance between the primary tumor and distant metastasis for T lymphocytes and Tregs was 15.4% and 50%, respectively. Changes in infiltration levels occurred in both directions; hence, no trend toward neither lower nor higher infiltration was observed (Table 5).

**Table 4:** Correlation between T lymphocyte (CD3+) infiltration and Treg (FOXP3+) infiltration in primary tumors, lymph node metastases, and distant metastases

		CD3 *Lnm*	CD3 *Dm*	FOXP3 *Prim*	FOXP3 *Lnm*	FOXP3 *Dm*
CD3 *Prim*	R	0,22	0,12	0,54	0,23	-0,24
	*p*	0,068	0,57	**0,000**	0,063	0,24
	N	69	26	172	68	26
CD3 *Lnm*	R		-0,39	0,26	0,55	0,23
	*p*		0,17	**0,031**	**0,000**	0,42
	N		14	70	78	14
CD3 *Dm*	R			-0,074	-0,47	0,61
	*p*			0,72	0,088	**0,000**
	N			26	14	34
FOXP3 *Prim*	R				0,19	-0,18
	*p*				0,12	0,37
	N				69	26
FOXP3 *Lnm*	R					-0,35
	*p*					0,22
	N					14

*Abbreviations*: *R* correlation coefficient, *N* number of patients included in analysis, *Prim* primary tumor, *Lnm* lymph node metastasis, *Dm* distant metastasis. Spearman correlati on, two-tailed *P* value. Bold indicates *P* value < 0.05

**Table 5:** Changes in infiltration levels of T lymphocyte (CD3+) and regulatory T lymphocytes (FOXP3+) over tumor progression

		*N*	Low → high	High → low	Discordance (%)	*P*
CD3	Prim → Lnm	69	10	7	24,6	0,63
	Lnm → Dm	14	1	2	21,4	1,0
	Prim → Dm	26	1	3	15,4	0,63
FOXP3	Prim → Lnm	69	19	12	44,9	0,28
	Lnm → Dm	14	4	4	57,1	1,0
	Prim → Dm	26	5	8	50,0	0,58

*Abbreviations*: *N* number of patients included in analysis, *Prim* primary tumor, *Lnm* lymph node metastasis, *Dm* distant metastasis. T lymphocytes (CD3) dichotomized into low (0 + 1 + 2) and high (3) infiltration. Regulatory T lymphocytes dichotomized into low (0) and high (1 + 2 + 3) infiltration. *P* value from McNemar’s test

### Treg infiltration in lymph node metastases

There was no prognostic impact of either T lymphocytes or Tregs in lymph node metastases (data not shown). However, some correlations to negative prognostic factors similar to those seen in primary tumors were shown for Tregs in lymph node metastases (see Additional file 5).

### Treg infiltration in distant metastases

**Fig. 4:**
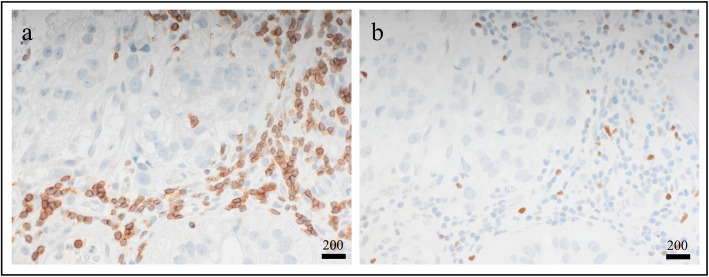
Immunohistochemical stainings of distant metastases. **a** CD3, a marker for T lymphocytes. **b** FOXP3, a marker for regulatory T lymphocytes. Scale bar = 200 μm

**Fig. 5:**
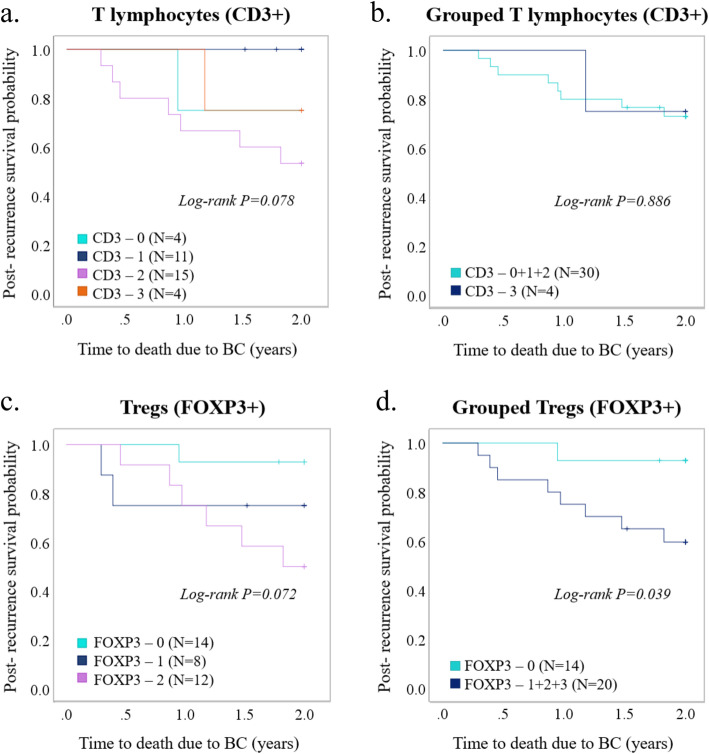
Prognostic role of Tregs in metastatic tumors. Kaplan-Meier plots (log-rank test) for post-recurrence survival depending on infiltration levels of **a** T lymphocytes (CD3^+^), **b** grouped T lymphocytes (CD3^+^), **c** regulatory T lymphocytes (Tregs, FOXP3^+^), and **d** grouped regulatory T lymphocytes (FOXP3^+^) in distant metastasis

We continued with exploring the potential clinical impact of T lymphocyte and Treg infiltration in distant metastases (Fig. 4; for distant metastasis sites, see Additional file 6). There were no correlations between either T lymphocyte or Treg infiltration and clinicopathological features related to distant metastases (see Additional file 7). However, while T lymphocyte infiltration in distant metastases showed no significant difference in 2-year post-recurrence survival (PRS) (*P =* 0.886) (Fig. 5a, b), there was a significant difference in PRS for Treg infiltration levels, with high infiltration being associated to worse outcome (*P* = 0.039) (Fig. 5c, d).

## Discussion

Previous studies have generally focused on the immune microenvironment in primary breast cancer tumors while less is known about the immune landscape in metastatic breast cancer and how it changes with metastatic progression. Distant metastases account for the great majority of deaths related to breast cancer and previous research has revealed highly varying post-recurrence survival once metastatic tumors are established [32, 33]. Despite advancements in diagnosing and treating breast cancer, the overall survival is approximately 75%, with global mortality rates differing profoundly and with metastatic disease accounting for the majority of breast cancer-related deaths [6, 34]. Importantly, immunotherapeutic alternatives have proven effective in treating advanced cancer; however, not all patients benefit from the treatment and as of yet immunomodulators have only been approved for some subsets of breast cancer patients [35, 36]. Deciphering which breast cancer patients with metastatic disease might benefit from immunotherapy could have important clinical implications. Here we reveal that Treg infiltration, in contrast to pan-T-lymphocyte infiltration, in distant metastases can identify those patients with worse 2-year survival and that accordingly might benefit from immunotherapies, e.g., CTLA-4-related therapeutics. CTLA-4 is an important immune checkpoint protein receptor and highly expressed on Tregs [37, 38]. Interestingly, ipilimumab, a fully humanized monoclonal antibody against CTLA-4, has been shown to reduce Treg infiltration in responders among melanoma patients [39].

The fact that high Treg infiltration in the metastatic tumor microenvironment correlated to a decreased 2-year PRS can be viewed as consistent with previous observations of the immunosuppressive properties of Tregs in aiding tumor progression seen in early breast cancer [11, 19–21]. The negative prognostic role of Tregs has also been shown in both early-stage breast cancer and locally advanced breast cancer [40, 41].

Moreover, we here show that high T lymphocyte and Treg infiltration in the primary tumor correlated to poor prognostic factors and shorter survival. T lymphocytes were shown to be an independent prognostic factor for RFS. This contradicts previous, although limited, studies showing a correlation between high T lymphocyte infiltration and better outcome [11–14]. The differential findings regarding the prognostic role of T lymphocyte infiltration in primary breast cancer might be that the cohort of this study only included patients having advanced stages of breast cancer. One could hypothesize that primary tumors in patients developing metastatic breast cancer recruit different T lymphocyte subtypes as compared to primary breast cancer in tumors lacking metastatic potential. Indeed, different subtypes are linked to different effects in the TME and prognostic outcome in cancer [42, 43]. Future longitudinal studies comparing the infiltration of various T lymphocyte subpopulations in primary tumors of patients who later develop or never develop metastatic breast cancer are needed to further clarify this. Our data also suggest that there are other T lymphocyte subpopulations, besides Tregs that could have important clinical implications in metastatic breast cancer. While the pan-T-lymphocyte marker CD3 was an independent prognostic factor for RFS, only FOXP3 was found to be an independent prognostic factor for BCSS, in line with previous work [19, 20], and able to decipher patients with worse 2-year PRS. These data suggest that other T lymphocyte subpopulations exist and have differential roles in metastatic breast cancer.

Previous studies have shown a decrease of total T lymphocyte and Treg infiltration from primary tumor to distant metastasis in breast cancer, with tumors becoming less immunogenic with progression [44–46]. Importantly, we could see a high discordance rate, especially in Treg infiltration. However, we observed changes in immune cell infiltrations in both directions. The fact that Treg, in particular, changes with metastatic progression illustrates the need to characterize metastatic breast tumors in detail independent of what is known about the immune landscape in the corresponding primary tumor.

Regarding other immune landscape markers assessed in primary tumors, we saw correlations between most immune cell populations. This can illustrate the important principal of what is referred to as “hot” and “cold” tumors, meaning that some tumors have a higher inflammatory activity than others and are more immunogenic [47]. The TN subtype has previously been shown to be the most immunogenic molecular subtype in breast cancer [48, 49] and is therefore currently viewed as being most appropriate for immunotherapeutic targeting [50]. Hence, a high infiltration of certain immune cell populations might not only be of negative prognostic value, but also predictive of positive response to immunotherapy. Interestingly, while Tregs and neutrophils correlated to TN subtype, and T lymphocytes, Tregs, macrophages, and neutrophils in primary tumors correlated to ER-negative breast cancer, no correlations to molecular subtype or other clinicopathological factors could be seen for either FOXP3 or CD3 in distant metastases, suggesting that Tregs in distant metastases could be a prognostic factor for patients with metastatic breast cancer independent of breast cancer subtype.

Strengths of this study include the large number of paired samples from primary tumors and lymph node metastases from patients with metastatic breast cancer, as well as the extensive information about clinicopathological factors that could be used in the analyses. To our knowledge, no previous study has shown the prognostic impact of immune cell infiltrations in distant metastases of breast cancer. As mentioned above, one important limitation was the limited material from metastatic locations. It would have been interesting to see whether certain immune profiles are associated to specific metastatic sites. Moreover, it would have been interesting to investigate if the prognostic role of Tregs in distant metastases is specific or whether other immune populations in metastatic breast cancer tumors also have prognostic impact. This would suggest that the concept of “hot” and “cold” tumors also applies to distant metastases. Future studies, preferentially including spatial profiling, are warranted to in more detail expand the understanding and the clinical impact of the immune microenvironment in metastatic breast cancer.

## Conclusions

Although the development of novel therapeutic alternatives has resulted in better outcome in the early stages of breast cancer, metastatic breast cancer still remains a clinical challenge with poor outcome. Developing novel therapies for metastatic disease and deciphering which patients might benefit from immunotherapy is therefore of great importance. In this study, we show that infiltration of T lymphocytes, Tregs, and macrophages in primary tumors is associated with worse outcome. Importantly, we also show that patients with high Treg infiltration in distant metastasis have shorter PRS and that Treg infiltration changes with metastatic progression.

## Supplementary Information

**Additional file 1.** Kaplan Meier plots (log-rank test) showing a,b. recurrence-free survival and c,d. breast cancer-specific survival among patients with different infiltration levels of Macrophages (CD68+) in primary tumor.

**Additional file 2.** Kaplan Meier plots (log-rank test) for a,b. recurrence-free survival and c,d. breast cancer-specific survival among patients with different infiltration levels of neutrophils (NE+) in primary tumor.

**Additional file 3.** Correlation between macrophage (CD68+) and neutrophil (NE+) immune cell infiltration and clinicopathological features in primary breast cancer.

**Additional file 4.** Correlation between immune cell populations within primary tumor.

**Additional file 5.** Correlation between T lymphocyte (CD3+) and regulatory T lymphocyte (FOXP3+) infiltration and clinicopathological features in lymph node metastasis.

**Additional file 6.** Location of distant metastases.

**Additional file 7.** Correlation between T lymphocyte (CD3+) and regulatory T lymphocyte (FOXP3+) infiltration and clinicopathological features in distant metastasis.

## Data Availability

Data sharing is not applicable to this article as no datasets were generated or analyzed during the current study.

## Abbreviations

TN: Triple negative
Treg: Regulatory T lymphocyte
ER: Estrogen receptor
TMA: Tissue microarray
IHC: Immunohistochemistry
PR: Progesterone receptor
RFS: Recurrence-free survival
BCSS: Breast cancer-specific survival
PRS: Post-recurrence survival
HR: Hazard ratio
