# Supplementary material for: Regulatory T lymphocyte infiltration in metastatic breast cancer—an independent prognostic factor that changes with tumor progression
Source: Breast Cancer Res. 2021 Feb 18;23:27. doi: 10.1186/s13058-021-01403-0 (PMC7893927; doi:10.1186/s13058-021-01403-0)
Supplement: Supplementary file 2 — Additional file 2. Kaplan Meier plots (log-rank test) for a,b. recurrence-free survival and c,d. breast cancer-specific survival among patients with different infiltration levels of neutrophils (NE+) in primary tumor. [file 13058_2021_1403_MOESM2_ESM.pdf]

Additional file 2

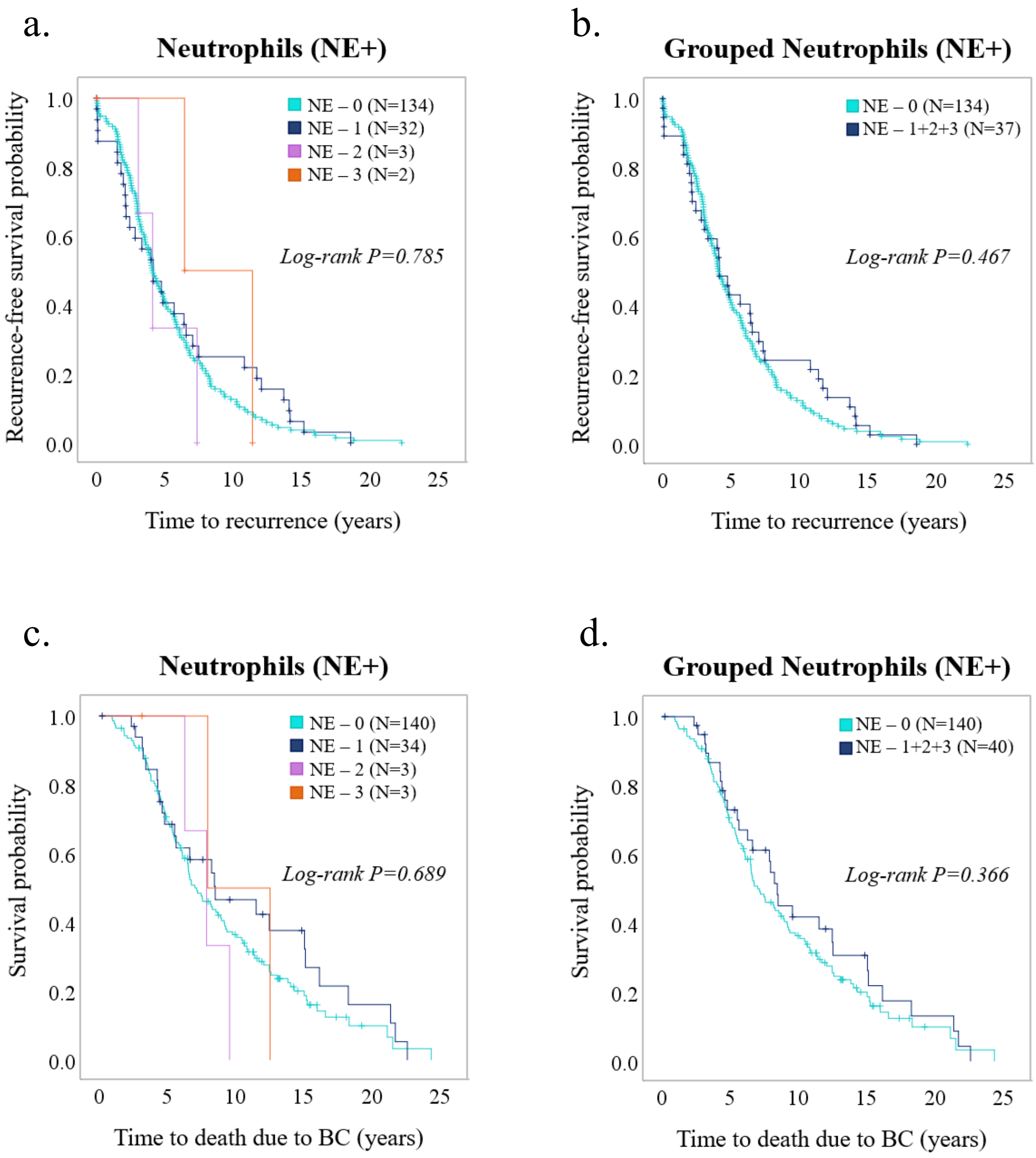

Additional file 2. Kaplan Meier plots (log-rank test) for **a,b.** recurrence-free survival and **c,d.** breast cancer-specific survival among patients with different infiltration levels of neutrophils (NE^+) in primary tumor.
