# Supplementary material for: Regulatory T lymphocyte infiltration in metastatic breast cancer—an independent prognostic factor that changes with tumor progression
Source: Breast Cancer Res. 2021 Feb 18;23:27. doi: 10.1186/s13058-021-01403-0 (PMC7893927; doi:10.1186/s13058-021-01403-0)
Supplement: Supplementary file 3 — Additional file 3. Correlation between macrophage (CD68+) and neutrophil (NE+) immune cell infiltration and clinicopathological features in primary breast cancer. [file 13058_2021_1403_MOESM3_ESM.pdf]

Additional file 3

Additional file 3. Correlation between macrophage (CD68$^+$) and neutrophil (NE$^+$) immune cell infiltration and clinicopathological features in primary breast cancer.

**Variable**	**N (%)**	**CD68 infiltration**				**R**	***P***	**N (%)**	**NE infiltration**				**R**	***P***
		**0**	**1**	**2**	**3**				**0**	**1**	**2**	**3**		
All	175 (100)	3	55	98	19			180 (100)	140	34	3	3		
Age														
<50	80 (45.7)	1	22	49	8	-0.061	0.42	83 (46.1)	59	22	1	1	-0.14	0.063
≥50	95 (54.3)	2	33	49	11			97 (53.9)	81	12	2	2		
Tumor size														
≤20 mm	72 (41.1)	1	25	39	7	0.051	0.51	75 (41.7)	59	15	1	0	0.029	0.70
>20 mm	102 (58.3)	2	30	58	12			104 (57.8)	80	19	2	3		
Nodal metastasis														
-	57 (32.6)	0	15	35	7	-0.085	0.27	59 (32.8)	44	14	0	1	-0.038	0.62
+	115 (65.7)	2	38	63	12			117 (65.0)	92	20	3	2		
Ki67														
-	110 (62.9)	3	35	63	9	0.12	0.12	109 (60.6)	91	17	1	0	0.16	**0.04**
+	56 (32.0)	0	15	32	9			62 (34.4)	44	14	2	2		
NHG														
Grade 1/2	65 (37.1)	2	23	37	3	0.14	0.074	64 (35.6)	51	11	1	1	0.033	0.68
Grade 3	89 (50.9)	1	26	48	14			94 (52.2)	72	20	1	1		
ER														
-	31 (17.7)	0	8	15	8	-0.15	**0.05**	33 (18.3)	20	9	2	2	-0.22	**0.004**
+	138 (78.9)	3	43	82	10			142 (78.9)	116	25	1	0		
PR														
-	71 (40.6)	1	23	35	12	-0.063	0.42	72 (40.0)	51	16	3	2	-0.16	**0.032**
+	97 (55.4)	2	29	61	5			102 (56.7)	85	17	0	0		
Luminal A	60 (34.3)	2	20	36	2	-0.17	**0.037**	62 (34.4)	54	8	0	0	-0.17	**0.027**
Luminal B	71 (40.6)	1	19	43	8	0.048	0.55	73 (40.6)	57	15	1	0	-0.003	0.97
HER2	7 (4.0)	0	0	6	1	0.13	0.11	9 (5.0)	6	3	0	0	0.062	0.43
TN	22 (12.6)	0	7	9	6	0.087	0.27	22 (12.2)	13	5	2	2	0.21	**0.007**

Abbreviations: N, number of patients included in analysis; R, correlation coefficient; Ki67, proliferation marker; NHG, Nottingham histologic grade; ER, estrogen receptor; PR, progesterone receptor; TN, triple negative. Spearman correlation, two-tailed *P*-value. Bold indicates *P*- value <0.05.
