# Supplementary material for: Regulatory T lymphocyte infiltration in metastatic breast cancer—an independent prognostic factor that changes with tumor progression
Source: Breast Cancer Res. 2021 Feb 18;23:27. doi: 10.1186/s13058-021-01403-0 (PMC7893927; doi:10.1186/s13058-021-01403-0)
Supplement: Supplementary file 4 — Additional file 4. Correlation between immune cell populations within primary tumor. [file 13058_2021_1403_MOESM4_ESM.pdf]

Additional file 4

Additional file 4. Correlation between immune cell populations within primary tumor.

		FOXP3	CD68	NE
CD3	R	0.54	0.43	0.20
	p	**0.000**	**0.000**	**0.007**
	N	172	170	173
FOXP3	R		0.34	0.053
	p		**0.000**	0.49
	N		172	174
CD68	R			0.23
	p			**0.003**
	N			172

Abbreviations: R, correlation coefficient; N, number of patients included in analysis. Spearman correlation, two-tailed *P*-value. Bold indicates *P*-value <0.05.
