# Supplementary material for: Regulatory T lymphocyte infiltration in metastatic breast cancer—an independent prognostic factor that changes with tumor progression
Source: Breast Cancer Res. 2021 Feb 18;23:27. doi: 10.1186/s13058-021-01403-0 (PMC7893927; doi:10.1186/s13058-021-01403-0)
Supplement: Supplementary file 5 — Additional file 5. Correlation between T lymphocyte (CD3+) and regulatory T lymphocyte (FOXP3+) infiltration and clinicopathological features in lymph node metastasis. [file 13058_2021_1403_MOESM5_ESM.pdf]

Additional file 5

Additional file 5. Correlation between T lymphocyte ($CD3^+$) and regulatory T lymphocyte ($FOXP3^+$) infiltration and clinicopathological features in lymph node metastasis.

Variable	N (%)	CD3 infiltration				R	*P*	N (%)	FOXP3 infiltration				R	*P*
		0	1	2	3				0	1	2	3		
All	81 (100)	5	21	40	15			80 (100)	26	36	18	0		
Age														
<50	36 (44.4)	2	11	18	5	0.10	0.38	36 (45.0)	13	13	10	0	-0.013	0.91
≥50	45 (55.6)	3	10	22	10			44 (55.0)	13	23	8	0		
Ki67														
-	52 (64.2)	5	12	26	9	0.048	0.68	51 (63.8)	22	21	8	0	0.30	**0.008**
+	25 (30.9)	0	8	12	5			26 (32.5)	4	13	9	0		
ER														
-	16 (19.8)	0	4	8	4	-0.12	0.29	17 (21.3)	4	8	5	0	-0.11	0.32
+	64 (79.0)	5	17	32	10			63 (78.8)	22	28	13	0		
PR														
-	42 (51.9)	2	10	20	10	-0.14	0.24	44 (55)	14	17	13	0	-0.12	0.30
+	34 (42.0)	3	9	18	4			33 (41.3)	11	18	4	0		
Luminal A	42 (51.9)	5	9	22	6	-0.062	0.59	42 (52.5)	18	17	7	0	-0.24	**0.037**
Luminal B	20 (24.7)	0	8	9	3	-0.040	0.73	19 (23.8)	4	10	5	0	0.13	0.27
HER2	4 (4.9)	0	2	2	0	-0.093	0.43	5 (6.3)	2	3	0	0	-0.10	0.38
TN	10 (12.3)	0	2	4	4	0.21	0.076	10 (12.5)	1	4	5	0	0.26	**0.021**

Abbreviations: N, number of patients included in analysis; R, correlation coefficient; Ki67, proliferation marker; ER, estrogen receptor; PR, progesterone receptor; TN, triple negative. Spearman correlation, two-tailed *P*-value. Bold indicates *P*- value <0.05.
