# Supplementary material for: Regulatory T lymphocyte infiltration in metastatic breast cancer—an independent prognostic factor that changes with tumor progression
Source: Breast Cancer Res. 2021 Feb 18;23:27. doi: 10.1186/s13058-021-01403-0 (PMC7893927; doi:10.1186/s13058-021-01403-0)
Supplement: Supplementary file 6 — Additional file 6. Location of distant metastases. [file 13058_2021_1403_MOESM6_ESM.pdf]

Additional file 6

Additional file 6. Location of distant metastases.

**Metastatic site**	**N**
Cutaneous/Subcutaneous	8
Bones	7
Liver	7
Distant lymph node metastases	3
Local	4
Brain	1
Ureter	1
Vagina	1
Muscle	1
Unknown	1

Abbreviations: N, number of patients.
