# Supplementary material for: Regulatory T lymphocyte infiltration in metastatic breast cancer—an independent prognostic factor that changes with tumor progression
Source: Breast Cancer Res. 2021 Feb 18;23:27. doi: 10.1186/s13058-021-01403-0 (PMC7893927; doi:10.1186/s13058-021-01403-0)
Supplement: Supplementary file 7 — Additional file 7. Correlation between T lymphocyte (CD3+) and regulatory T lymphocyte (FOXP3+) infiltration and clinicopathological features in distant metastasis. [file 13058_2021_1403_MOESM7_ESM.pdf]

Additional file 7

Additional file 7. Correlation between T lymphocyte ($CD3^+$) and regulatory T lymphocyte ($FOXP3^+$) infiltration and clinicopathological features in distant metastasis.

Variable	N (%)	CD3 infiltration				R	*P*	N (%)	FOXP3 infiltration				R	*P*
		**0**	**1**	**2**	**3**				**0**	**1**	**2**	**3**		
All	34 (100)	4	11	15	4			34 (100)	14	8	12	0		
Age														
<50	19 (55.9)	3	7	7	2	0.19	0.29	19 (55.9)	7	5	7	0	-0.078	0.66
≥50	15 (44.1)	1	4	8	2			15 (44.1)	7	3	5	0		
Ki67														
-	17 (50.0)	1	8	7	1	0.072	0.74	17 (50.0)	5	6	6	0	-0.028	0.90
+	7 (20.6)	1	2	3	1			7 (20.6)	3	1	3	0		
ER														
-	9 (26.5)	1	4	3	1	0.035	0.87	9 (26.5)	3	3	3	0	0.034	0.87
+	17 (50.0)	2	6	8	1			17 (50.0)	6	4	7	0		
PR														
-	21 (61.8)	2	9	8	2	-0.01	0.96	21 (61.8)	8	5	8	0	0.00	1.00
+	3 (8.8)	1	0	2	0			3 (8.8)	1	1	1	0		
Luminal A	12 (35.3)	1	4	6	1	0.20	0.38	12 (35.3)	4	3	5	0	-0.008	0.97
Luminal B	3 (8.8)	0	2	1	0	-0.091	0.69	3 (8.8)	1	1	1	0	-0.044	0.84
HER2	2 (5.9)	1	0	1	0	-0.14	0.55	2 (5.9)	1	0	1	0	-0.027	0.91
TN	5 (14.7)	0	3	2	0	-0.065	0.77	5 (14.7)	1	2	2	0	0.064	0.78

Abbreviations: N, number of patients included in analysis; R, correlation coefficient; Ki67, proliferation marker; ER, estrogen receptor; PR, progesteron receptor; TN, triple negative. Spearman correlation, two-tailed *P*-value. Bold indicates *P*- value <0.05.
